# Bilateral Vocal Cord Paralysis Secondary to Leptomeningeal Metastases With Unknown Primary Malignancy: A Case Report and Review of the Literature

**DOI:** 10.7759/cureus.27425

**Published:** 2022-07-28

**Authors:** Jennifer Chen, Phillip Staibano, Kelvin Zhou, Michael Gupta

**Affiliations:** 1 Otolaryngology—Head and Neck Surgery, McMaster University, Hamilton, CAN; 2 Otolaryngology–Head and Neck Surgery, McMaster University, Hamilton, CAN

**Keywords:** otolaryngology, head and neck pathologies, neurology, leptomeningeal carcinoma, bilateral vocal cord paralysis

## Abstract

Bilateral vocal cord paralysis (BVCP) most commonly occurs secondary to iatrogenic injury and/or malignancy, but can also be a consequence of central nervous system (CNS) pathology. We report a case of BVCP secondary to leptomeningeal consequence in the context of unknown primary malignancy. The aim of this report is to promote awareness for BVCP caused by rare CNS pathology and highlight the importance of complete neoplastic and paraneoplastic workups in new-onset BVCP with unclear etiology. Here, we present a case report and review of the literature. A 68-year-old female presented with new-onset BVCP in the context of progressive dysphagia in addition to rectal and urinary incontinence. She underwent an awake tracheostomy. Her infectious and paraneoplastic workups did not identify a cause for her BVCP. Her brain MRI demonstrated enhancement of multiple cranial nerves, spine MRI demonstrated leptomeningeal enhancement, and cerebrospinal fluid (CSF) cytology was positive for metastatic adenocarcinoma. Her functional status was poor and she was deemed ineligible for chemotherapy and transitioned to palliative care. She died three months following her hospital admission. Leptomeningeal metastasis is a rare cause of new-onset BVCP. Airway management remains a critical component in BVCP. The sudden onset of BVCP in the context of generalized neurologic symptoms or cranial nerve deficits should prompt complete neoplastic and paraneoplastic investigation.

## Introduction

Bilateral vocal cord immobility arises from restricted vocal fold movement secondary to neurological or mechanical etiology [1]. In terms of neurological etiology, defined as bilateral vocal cord paralysis (BVCP), the most common etiologies are iatrogenic or malignancy but it can also occur due to systemic disease, and/or central nervous system (CNS) pathology. Leptomeningeal metastasis (LM), otherwise termed leptomeningeal carcinomatosis, is a rare complication of malignancy that occurs via the invasion of the leptomeninges by cancer cells [2]. An estimated 3-8% of advanced solid tumors, including breast, lung, gastrointestinal, melanoma, and primary CNS tumors, can lead to LM [3]. Leptomeningeal metastasis secondary to an unknown primary malignancy is a rare clinical entity [4]. Cytological examination of cerebrospinal fluid (CSF) remains the gold standard for diagnosing LM, but neuroimaging modalities such as MRI are being more readily employed due to notable improvements in its diagnostic sensitivity [2]. Prognostically, LM is associated with a median survival of two to four months and treatment is typically supportive with a goal of improving quality of life and extending survival. Additional treatment can include radiotherapy to symptomatic lesions, systemic chemotherapy, and/or intrathecal chemotherapy, which has demonstrated the most notable survival benefit in LM [5].

We report, to our knowledge, the first case report of sudden-onset BVCP secondary to leptomeningeal metastasis in the context of an unknown primary malignancy. This diagnosis of LM was made in the context of both neuroimaging and gold standard CSF cytology. This report highlights the importance of performing a full neoplastic, paraneoplastic, and neurological work in the presence of new-onset BVCP of unknown etiology.

## Case presentation

A 68-year-old female presented with a two-week history acute-on-chronic exertional dyspnea and inspiratory stridor. Otherwise, she had a subjective history of dysphagia to both solids and liquids and a 40-pound weight loss. Moreover, she reported a six-month history of nausea and vomiting with reduced oral intake. She did report a number of repeated falls and a gradual loss of independent mobility, multiple episodes of rectal and urinary incontinence, and saddle anesthesia in the months prior to presentation. 

She had a medical history significant for adenoid cystic carcinoma of her lip that was treated with primary surgical resection. She also had a history of uterine serous adenocarcinoma treated four years prior via total abdominal hysterectomy and bilateral salpingo-oophrectomy, in addition to adjuvant radiation and chemotherapy. She was also noted to have unresectable nodal disease, but was not undergoing any active cancer treatment at time of presentation.

**Figure 1:**
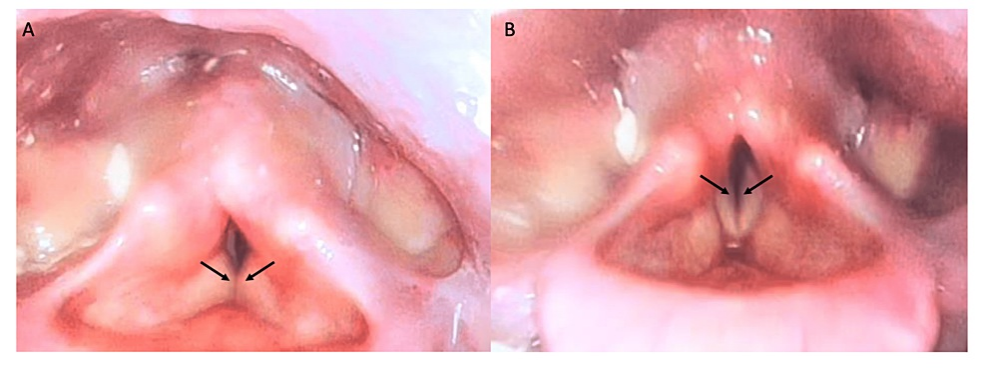
(A) Flexible nasopharyngolaryngoscopy with black arrows demonstrating plica ventricularis during phonation. (B) Flexible nasopharyngolaryngoscopy with black arrows demonstrating true vocal cords fixed within the paramedian position during inspiration.

Her physical examination demonstrated O2 saturation of 92% on one litre of oxygen, intermittent inspiratory stridor, but no overt work of breathing. Flexible nasopharygolaryngoscopy (NPL) demonstrated bilateral vocal cord immobility (BVCI) and intermittent plica ventricularis (Figure 1A). Her vocal cords were in the paramedian position bilaterally with a 3 mm midline-posterior glottic gap (Figure 1B). Her neurological exam demonstrated poor anal sphincter tone, in addition to 3/5 strength in both her upper and lower extremities. Her cranial nerve, head and neck, and abdominal exams were unremarkable.

On admission, her MRI of her lumbar spine was negative for cauda equina syndrome. Her chest x-ray demonstrated hyperinflation and she was started on management for a presumed chronic obstructive pulmonary disease (COPD) exacerbation. Her CT neck and chest did not demonstrate any lesions underlying her BVCI or inspiratory stridor. Her venous blood gases were suggestive of a compensated respiratory acidosis, but her infectious blood work was unremarkable. She underwent an open tracheostomy and panendoscopy. Intraoperatively, she had mobile cricoarytenoid joints and no mucosal lesions were identified. Her investigations for adrenal insufficiency to explain her ongoing weight loss were negative. She had a negative CT chest/abdomen/pelvis, and an unremarkable colonoscopy and esophagoscopy, helping to rule against a paraneoplastic etiology for her BVCI. Moreover, she underwent a diagnostic bronchoscopy that demonstrated bronchial alveolar lavage and right upper lobe biopsy that were negative for malignancy. Her autoimmune workup was negative and her thyroid function was unremarkable.

**Figure 2:**
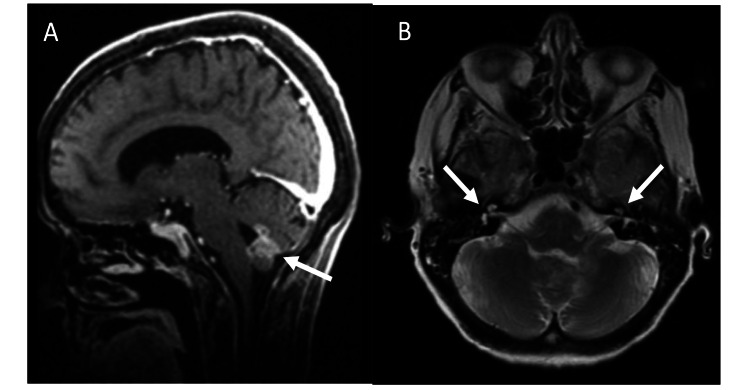
MRI brain with gadolinium (A) Sagittal image of T1 brain MRI with gadolinium with white arrows demonstrating heterogeneously enhancing lesion at the area postrema. (B)  Axial image of T2 brain MRI with gadolinium with white arrows demonstrating enhancement of cranial nerves VII and VIII.

She was subsequently evaluated by the neurology service for this constellation of symptoms and was found to have hyporeflexia and new-onset right ptosis, but otherwise no focal cranial nerve deficits. She was found to have a non-functional swallow and underwent percutaneous gastrostomy tube. Her additional neurological workup included a lumbar puncture with final cytology demonstrating metastatic adenocarcinoma. A brain MRI with gadolinium demonstrated a heterogeneously enhancing lesion centered within the dorsal fourth ventricle and inferior vermis with involvement of the area postrema, in addition to enhancement of multiple cranial nerves (Figure 2). A subsequent spine MRI with gadolinium demonstrated nodular leptomeningeal enhancement consistent with leptomeningeal disease. After discussion with the patient, she did not wish to undergo any additional investigations, including whole-body positron emission tomography (PET) imaging to further evaluate for the site of primary malignancy. 

In collaboration with neuro-oncology service and family, she was deemed ineligible for chemotherapy due to her worsening functional status. She was transitioned to palliative measures and died peacefully two months following her hospital admission.

## Discussion

To the best of our knowledge, this is the first case report demonstrating LM, diagnosed via radiographic and cytologic evidence, as a central etiology for new-onset BVCP. As mentioned previously, BVCP can be secondary to a number of etiologies, including malignancy, iatrogenic, and autoimmune disease (Table 1). One previous case report has demonstrated BVCP as a consequence of LM, but this report did not confirm diagnosis via CSF cytology [6]. Leptomeningeal metastasis commonly presents with vague neurologic symptoms; however, it can manifest with focal sensory deficits including hearing and vision loss [7]. In this case, clinical manifestations of potential cranial nerve deficits included BVCP and ptosis, while brain MRI demonstrated enhancement of multiple cranial nerves. Cranial nerve palsies, including those affecting the facial and vestibulocochlear nerve, have been reported to occur as a consequence of LM [8]. In the setting of LM, hematologic malignancies are most commonly associated with the development of cranial nerve deficits, while their onset is suggestive of poor prognosis and do not typically respond to treatment [6,9].

**Table 1:** Differential diagnosis of bilateral vocal cord paralysis (BVCP)

Etiology	Mechanism of BVCP	Examples
Iatrogenic	Scarring of the vocal cords or injury to the nerves that supply the vocal cords	Prolonged intubation, surgical trauma to the recurrent laryngeal nerve
Malignancy	Destruction of the vocal cords, associated joints, and/or neural invasion	Laryngeal malignancy, subglottic malignancy, thyroid malignancy
Central nervous system (CNS) pathology	Disruption of upper motor neuron pathways	Stroke, central nervous system neoplasm, multiple sclerosis
Systemic disease	Disruption of upper motor neuron or lower motor neuron pathways	Amyotrophic lateral sclerosis, Miller-Fisher syndrome
Idiopathic	--	--

Despite a complete malignancy workup, we were unable to identify a primary malignancy to explain this development of LM, which is consistent with the literature suggesting that LM with an unknown primary malignancy remains a rare clinical entity [4]. One possible reason for LM onset in this case was the previous history of advanced serous uterine adenocarcinoma, has been shown, in rare instances, to be associated with LM [10]. In gynecologic cancers, LM is most often associated with ovarian cancers with few cases occurring secondary to cervical and endometrial carcinoma. We recommend that a complete panel of neoplastic and paraneoplastic investigations should be performed in all patients who present with BVCP of unknown etiology, especially in patients with neuroimaging suggestive of CNS pathology. Although not performed in the presented case report, we do recommend considering full body PET imaging in order to further investigate the location of the primary malignancy. 

## Conclusions

In conclusion, BVCP is a rare consequence of LM, especially in the context an unknown primary malignancy. We propose that patients with BVCP and non-specific neurologic symptoms with a history of solid tumor malignancy warrant prompt diagnostic workup with cytological examination and neuroimaging for CNS pathology.
